# Utilizing a Prototype Patient-Controlled Electronic Health Record in Germany: Qualitative Analysis of User-Reported Perceptions and Perspectives

**DOI:** 10.2196/10411

**Published:** 2018-08-03

**Authors:** Regina Poss-Doering, Aline Kunz, Sabrina Pohlmann, Helene Hofmann, Marion Kiel, Eva C Winkler, Dominik Ose, Joachim Szecsenyi

**Affiliations:** ^1^ Department of General Practice and Health Services Research University Hospital Heidelberg Heidelberg Germany; ^2^ Ethics and Patient-Oriented Care National Centre for Tumor Diseases Heidelberg Germany; ^3^ Division of Cancer Population Science Department of Population Health Services University of Utah Salt Lake City, UT United States

**Keywords:** personal patient-controlled electronic health record, eHealth, nationwide implementation, continuity of care

## Abstract

**Background:**

Personal electronic health records (PHR) are considered instrumental in improving health care quality and efficiency, enhancing communication between all parties involved and strengthening the patient’s role. Technical architectures, data privacy, and applicability issues have been discussed for many years. Nevertheless, nationwide implementation of a PHR is still pending in Germany despite legal regulations provided by the eHealth Act passed in 2015. Within the information technology for patient-oriented care project funded by the Federal Ministry of Education and Research (2012-2017), a Web-based personal electronic health record prototype (PEPA) was developed enabling patient-controlled information exchange across different care settings. Gastrointestinal cancer patients and general practitioners utilized PEPA during a 3-month trial period. Both patients and physicians authorized by them could view PEPA content online and upload or download files.

**Objective:**

This paper aims to outline findings of the posttrial qualitative study carried out to evaluate user-reported experiences, perceptions, and perspectives, focusing on their interpretation of PEPA beyond technical usability and views on a future nationwide implementation.

**Methods:**

Data were collected through semistructured guide-based interviews with 11 patients and 3 physicians (N=14). Participants were asked to share experiences, views of perceived implications, and perspectives towards nationwide implementation. Further data were generated through free-text fields in a subsequent study-specific patient questionnaire and researcher’s notes. Data were pseudonymized, audiotaped, and transcribed verbatim. Content analysis was performed through the Framework Analysis approach. All qualitative data were systemized by using MAXQDA Analytics PRO 12 (Rel.12.3.1). Additionally, participant characteristics were analyzed descriptively using IBM SPSS Statistics Version 24.

**Results:**

Users interpreted PEPA as a central medium containing digital chronological health-related documentation that simplifies information sharing across care settings. While patients consider the implementation of PEPA in Germany in the near future, physicians are more hesitant. Both groups believe in PEPA’s concept, but share awareness of concerns about data privacy and older or impaired people’s abilities to manage online records. Patients perceive benefits for involvement in treatment processes and continuity of care but worry about financing and the implementation of functionally reduced versions. Physicians consider integration into primary systems critical for interoperability but anticipate technical challenges, as well as resistance from older patients and colleagues. They omit clear positioning regarding PEPA’s potential incremental value for health care organizations or the provider-patient relationship.

**Conclusions:**

Digitalization in German health care will continue to bring change, both organizational and in the physician-patient relationship. Patients endorse and expect a nationwide PEPA implementation, anticipating various benefits. Decision makers and providers need to contribute to closing modernization gaps by committing to new concepts and by invigorating transformed roles.

## Introduction

Personal electronic health records (PHR) and patient access to them have been discussed for quite some time. Since Shenkin and Warner [1] proposed that patients should have complete access to their medical records in 1973, supporting arguments have been confirmed in multiple studies and stand unaltered. This has led to (1) improved doctor-patient communication, (2) patient empowerment and education, (3) increased understanding of treatment plans, and (4) therapy adherence [2-5].

German law entitles patients to review their medical records and request paper or electronic copies of documents detailing their care processes [6]. However, there is no structured exchange of information beyond the doctor’s written reports [7] and the majority of health documentation is retained by the treating physician or hospital. While few primary care practices still use paper records, others have long since introduced electronic systems for documentation and administrative purposes. These primary systems contain patient records that are often inaccessible and lacking in health history documentation control. Information exchange between health care providers is often done by mail or fax, and sometimes even by the patients themselves or family members [7].

In December 2015, the passing of the Act for Secure Digital Communication and Applications in the Health Sector (eHealth Act) [8,9] laid the legal groundwork for an electronic exchange of health-related documentation for all patients in Germany. This law promotes the entry point for a PHR since prerequisites for a secure digital infrastructure now are due to be in place by the end of 2018. From then on, digital patient-related data like physician reports, emergency, and medication information can be made available in a PHR, enabling patients to access their data and inform providers about their medical history [9]. However, the type of record has yet to be determined.

**Figure 1:**
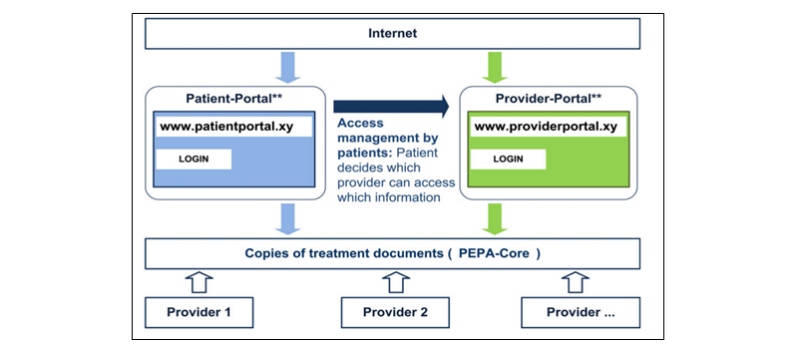
The PEPA concept [13].

The Web‐based personal electronic health record prototype (PEPA) developed within information technology for patient-oriented care (INFOPAT) differs from institution-related solutions moderated by health care personnel. Based on previously determined and integrated user requirements [10,11] and explored perceived benefits and concerns [12], its’ unique user-centered design facilitates a patient-controlled Web-based exchange of information across different care settings and providers (Figure 1 adapted from [13]). It understands patients as active participants in the care process [13] and enables them to manage which providers can access their medical documentation.

PEPA’s concept comprises a patient portal as well as a professional portal. For a 3-month trial period, PEPA was implemented into a real-world regional care setting to be utilized by gastrointestinal cancer patients and general practitioners (GPs), with the outpatient clinic at the National Center for Tumor Diseases (NCT) being involved as a cancer treatment facility. Patients and their authorized physicians could view PEPA content online and perform uploads and downloads of files, including doctor’s reports, laboratory, and imaging results. Sharing information and communication between all involved has been identified to be among the specific challenges of care delivery to this particular patient collective [14].

Previous studies focused on recurring themes of data privacy, functionality, expectations or identifying barriers to adopting electronic solutions [12,15-17]. The purpose of this posttrial qualitative study was to look beyond the factors mentioned above to better understand user-reported experiences which are integral to efforts of refinement. To answer the question which insights could be gained from utilizing the prototype, the reported overall experiences were evaluated with a focus on users’ interpretations of PEPA, perceived implications, and views on a potential future nationwide implementation.

## Methods

### Overview

Following the technical development of PEPA, the prototype was exclusively implemented into a real-world regional health care setting. After receiving tailored training, enrolled participants used the patient portal to upload and download personal health documentation, access linked certified educational information, and authorize others to add, and view content. Participating health care providers could use the professional portal to upload medical documentation related to the respective patient and read files if patients had granted access. All users could access and utilize the portals until the prototype was discontinued at the end of November 2016. The study was approved by the Ethics Committee of the University of Heidelberg (S-462-2015). Participants all gave written informed consent. Confidentiality and anonymity were ensured throughout the study.

### Study Design

As defined by the study protocol [13], a posttrial qualitative study was conducted to evaluate user-reported experiences and perceptions, using semistructured guide-based interviews with 11 gastrointestinal cancer patients, and 3 physicians. To ensure a broad perspective, the interview guide was developed by an interprofessional team of researchers (social scientists, physician, health scientist). It was designed to explore participants’ interpretations of PEPA, how and whether involvement in care was affected, behavioral and emotional experiences resulting from utilizing PEPA, assessment of training and support, and to gain insights into their perspectives regarding a future nationwide implementation. Also, participants were required to fill in a survey after the interview at the end of the trial period and return it by mail.

### Sampling and Recruitment

No formal sample size was calculated. Between July 25 and August 25, 2016, a random sample of 17 gastrointestinal cancer patients was recruited through NCT and the INFOPAT study team (Department of General Practice and Health Services Research, University Hospital Heidelberg). Potential participants had to be in ongoing therapy at NCT with a diagnosis of colorectal cancer (ie, C18, C19, or C20), or other gastrointestinal tumor diseases (ie, C16, C23.9, C24.0, C24.1), be at least 18 years old, and legally fully competent. Other prerequisites were a fluent command of German, access to a computer with internet connection, and participation in the study-specific training. Patients with severe acute psychiatric disorders, dementia, and behavioral or psychological disorders after consuming psychoactive substances were excluded. Since the sample consisted of gastrointestinal cancer patients—some with a limited diagnosis—recruitment followed a structured screening procedure conducted by an oncologist and included a thorough assessment of the patients’ condition and status prior and during recruitment efforts as well as their confirmed interest in participating [13]. Only the 17 patients who met all defined criteria in the recruitment month were included in the study, received printed material, and were provided with additional information over the phone.

Following the individual training sessions, all 17 patients were asked if they thought their GP would like to join the study and could be approached by the study team. Pursuing a purposive sampling strategy, recruitment letters were mailed to 15 GP practices, supplemented by detailed background information. Follow-up calls outlined study goals and procedures, the 3-month-long trial phase and PEPA’s concept (Figure 1). Five GPs expressed interest, but 2 had to be excluded due to technical challenges. Three GPs were recruited. Two were trained and used PEPA’s professional portal during the trial phase. The third GP did not receive training as the patient no longer required treatment at NCT at the time of the scheduled session. Identified challenges that led to the small number of participating GPs will be reported separately.

A total of 13 patients filled in the posttrial survey which was designed as a composite of German versions of validated measurement instruments (see Table 1, adapted from [13]) and gave room for free text. Based on their condition or passing away during the trial period, 6 patients could not be interviewed and were lost to the sample. No substitute patients could be recruited. Eleven patients (5 male, 6 female) utilized PEPA and participated in the interview and the posttrial survey. They ranged in age from 27 to 64 years. The physician age ranged from 29 to 58 and all 3 were female. All participants gave written informed consent for the study and audio recording of the interview and received a small reimbursement for their participation.

### Data Collection and Analysis

Table 2 summarizes the data collection sources. All interviews were conducted and audio recorded between November 2016 and January 2017 by researchers of the study team. Patient interview duration ranged from 37-82 minutes, with a mean of 50 minutes. Physician interviews lasted between 30-42 minutes, with a mean of 36 minutes. To accommodate a patient’s request, a spouse was present during 1 interview. All patient interviews and the first physician interview were conducted face-to-face at the Department of General Practice and Health Services Research of the University Hospital Heidelberg. The second physician interview was performed over the telephone. The third took place at the GPs practice. Additional notes were taken during and after 5 interviews.

**Table 1:** Compilation of the posttrial survey.

Outcome parameter	Outcome measurement instrument	# of items
Patient self-efficacy	Cancer Behavior Inventory Brief German Version [18,19]	14
Involvement in care	Perceived Involvement in Care Scale [20,21]	13
Psychosocial distress	Distress Management Thermometer [22,23]	1
Control preferences	Control Preferences Scale [24]	5
Usability of PEPA prototype	System Usability Scale [25,26]	10
Utilization of medical services	Mannheimer Module Resource Consumption^a^	30

^a^Not published.

**Table 2:** Source of data collection for this study (N=14).

Source of data collection	Patients	Physicians	Description of data source
Number of interviews conducted, n (%)	11 (78)	3 (22)	Face-to face and telephone interviews
Interview duration (minutes), mean (range)	50 (37-82)	36 (30-42)	Audio files and transcripts
Surveys conducted, n (%)	11 (100)	—	Free text, after interview and participant characteristics
Researcher’s notes, n (%)	1 (20)	4 (80)	Notes taken during and after interviews

**Table 3:** Translated interview guide used to conduct the qualitative interviews with patients and physicians.

Question or stimulus		Addressed to:	
		Patient	Physician
**Relate your experience of utilizing PEPA to its significance for you regarding:**			
	Your medical condition	Yes	—
	The provider-patient-relationship	—	Yes
Talk about how and how often you used PEPA		Yes	Yes
What has been positive or negative from your perspective?		Yes	Yes
**Tell us about changes you registered during your use of PEPA with regards to:**			
	Disease-specific knowledge and health literacy	Yes	—
	Provider-patient dialogue and general communication	Yes	Yes
	Being involved in care processes	Yes	—
In hindsight, what can you tell about the training session and support?		Yes	Yes
Did you experience any distress or anxiousness related to using PEPA?		Yes	—
Did you experience any distress or difficulties using PEPA?		—	Yes
Thought experiment: Which aspects should a friend consider if given the chance to utilize PEPA? Which advice would you provide?		Yes	—
Which chances or obstacles do you see for intersectoral collaboration?		—	Yes
**What is your perspective on a nationwide PEPA implementation regarding:**			
	Potential users, additional functionality, chances, and obstacles?	Yes	Yes
	Integration into existing care process?	Yes	Yes
	Appropriate support activities?	—	Yes
What would you like to tell us besides already discussed topics?		Yes	Yes
What was your motivation for participation in the study?		Yes	Yes

**Figure 2:**
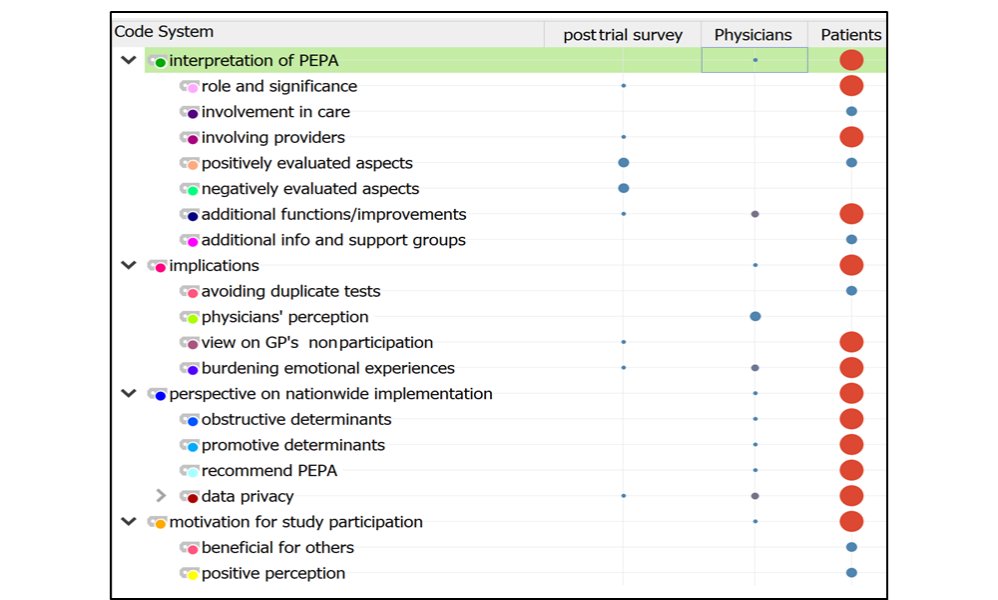
The thematic analysis framework reflected by the code system matrix. GP: general practitioner.

After data collection was completed, verbatim transcripts were coded using the matrix-based method of Framework Analysis [27-29] which is seen as an appropriate content analysis approach in a study with predetermined research questions [30]. Themes of interest were identified deductively a priori from the interview guide (Table 3) as well as inductively de novo from the data during the analysis. Transcripts, researchers’ additional notes, and free texts and comments given in the survey were coded iteratively using MAXQDA Analytics PRO 12 (Release 12.3.1). To enable a broader view, participant characteristics and selected items of the posttrial survey data were analyzed descriptively by using IBM SPSS Statistics Version 24.

Adequate methodological strategies were followed to ensure the trustworthiness of the analysis and findings. These include seeking out similarities and differences across and within accounts to ensure different perspectives are represented, as well as engaging with other researchers to minimize research bias, thus reducing the risk of losing relevant content.

Charting participant views concerning identified themes enabled comparisons within and across interviews [30] thus enhancing the transparency of the analysis [29]. The code system matrix reflects the thematic framework for the analysis (Figure 2). Here the symbol size indicates the proportional distribution of themes in the 3 document groups.

## Results

### Overview

**Figure 3:**
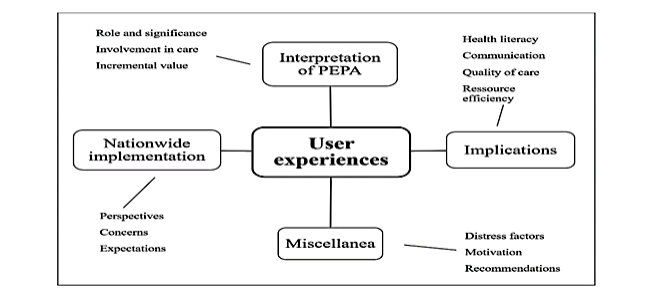
Overview of identified principal results of user experiences.

The primary results outlined broadly reflect the thematic spectrum of PEPA user experiences (Figure 3). Participant characteristics provide further indication referring to user perceptions (Table 4). Findings are presented in categories, subcategories, and key aspects and are differentiated by user group where applicable (Table 5). Quotes extracted from the data and cited here were translated into English with due diligence.

### User’s Interpretation of the Web‐Based Personal Electronic Health Record and its Role

Some thematic aspects were common to all 11 participating patients. PEPA was interpreted as a well-functioning administrative documentation system and regularly utilized to follow up on care processes. A frequently described benefit was keeping the chronological health-related digital documentation in a central, easily accessible medium that facilitates and simplifies data sharing across care settings. Emphasis was also put on the importance of the prompt availability of documentation which could make paper documentation obsolete.

The advantage is, I think, to have everything consolidated, I don’t have to search for everything and ask for MRI images somewhere or a doctor’s report, but I have a chronological history where I have instant access and, in this respect, I think it would be an asset for doctors as well.

Patient #21, male

A few patients were skeptical of system maintenance reliability, and therefore 2/11 (18%) still asked for paper copies of documents to maintain their paper files. Focusing on future needs, 4 (36%) patients developed a problem-oriented strategy and produced electronic copies to keep on their electronic storage devices or even turned to a different cloud solution for file sharing during the trial phase.

**Table 4:** Outline of the participant characteristics (N=14).

Characteristics		Patients (n=11)	Physicians (n=3)
Participant disease or physician specialty		Gastrointestinal cancer	General practitioner, oncologist
**Gender, n (%)**			
	Female	6 (55)	3 (100)
	Male	5 (45)	—
Age (years), range		27-64	29-58
**Age (years), mean**			
	Female	47 (10.9)	42 (11.9)
	Male	57 (5.0)	—
Frequent internet user, n (%)		9 (85)	—
Internet connection at home only, n (%)		4 (36)	—
Mobile and at home, n (%)		7 (64)	—
Researching health topics on internet, n (%)		8 (73)	—
Confident when using PEPA, n (%)		10 (91)	—
Classified PEPA as easy to use, n (%)		9 (82)	—
Need expert support to use PEPA, n (%)		1 (9)	—

All 3 participating physicians rated PEPA positively and reported having used the system mainly to upload and share lab results or to look for new reports. They interpreted PEPA as a structured well-functioning documentation medium with the potential of improving communication between health care providers and patients as well as across different care settings.

Resembling patients’ views, both GPs attributed an incremental value to having fast access to structured information essential during the care process. Physicians also addressed the importance of provider-patient dialog.

What gets the patient ready for therapy preparation certainly is the dialog with the treating physician.

Physician #15, female

### Involvement in Care

Utilizing PEPA, patients felt more involved in care processes and expressed their hope for a more wholesome view of the patient via an extensively functioning PEPA. However, 5 (45%) patients stated disappointment about their GP not participating in the study and saw this as a break in the continuity of care. They speculated about potential reasons, expressed understanding, or saw missed opportunities for change and felt deprived of a very important user of the system.

**Table 5:** Overview of findings regarding patient and physician experiences and perspectives.

Category and aspect of the experience				User group
**Interpretation**				
	**Role and significance**			
		PEPA facilitates central documentation	Patients, physicians	
		Easy access and sharing	Patients, physicians	
		Makes paper obsolete	Patients, physicians	
		Improving communication	Physicians	
	**Involvement in care**			
		Patient takes control	Patients	
		More involved	Patients	
		Hope for wholesome view	Patients	
		Provider-patient dialog	Physicians	
	**Incremental value**			
		Fast access to structured data	Patients, physicians	
**Implications**				
	**Health literacy**			
		Engaging with documentation	Patients	
		Preparation and follow-up	Patients	
		Potential promoting factor	Patients	
	**Communication**			
		Face-to-face important	Patients	
		Faster communication	Patients, physicians	
	**Resource efficiency**			
		Reduction of expenditures	Patients	
		Economy of time	Physicians	
	**Quality of care**			
		Transparency of documentation	Physicians	
		Patient safety	Physicians	
		Optimization of care processes	Patients, physicians	
**Implementation**				
	**Perspectives**			
		Implementation realistic	Patients	
		Life-long usage	Patients	
	**Concerns**			
		Presumed non-acceptance	Patients, physicians	
		Data privacy	Patients, physicians	
		Functionally reduced systems	Patients, physicians	
	**Expectations**			
		Obligatory for general practitioners	Patients	
		Integration into primary system	Physicians	
		Misuse improbable	Patients, physicians	
**Miscellanea**				
	**Distress factors**			
		Emotional distress excluded	Patients	
		Influence of individual factors	Patients, physicians	
		Provider-patient dialog important	Patients, physicians	
		Keep personal notes inaccessible	Physicians	
	**Recommendations**			
		Careful access authorization	Patients	
		Advice to use PEPA	Patients, physicians	
	**Motivation**			
		Intent to contribute to research	Patients, physicians	
		Help others through input	Patients, physicians	
		Learning opportunity	Physicians	
		Varying perceptions of own role	Patients, physicians	

One (9%) patient reported the GP was not involved in the cancer care at all.

Let’s say, as a patient one would take over a little more control. …I think you tend to inform yourself more thoroughly, perhaps.

Patient #21, male

My GP simply could have uploaded my lab results into the PEPA portal for NCT to look at them the day of my chemo. He didn’t do it, so he had to send them by fax.

Patient #27, female

### Implications of Utilizing the Web‐Based Personal Electronic Health Record

With regards to dealing with their illness, 6 (55%) patients generally did not attribute strong significance to PEPA or perceive a difference to receiving paper documentation. After being confronted with the illness for quite some time already, they felt empowered by disease-specific knowledge and confident to objectively classify the report content. However, they reported that utilizing PEPA made engaging with health documentation easier, considered it valuable for preparation and follow-up of appointments, and saw the potential for health literacy aspects.

I don’t believe utilizing PEPA will lead to somehow increased literacy, just by using it, but it could be developed into this direction.

Patient #06, male

Nearly half (5/11, 45%) of the patients addressed communication aspects and the provider-patient relationship by pointing out the importance of having a face-to-face conversation with their physician about their status or new findings before reports would be uploaded into PEPA. Also, they voiced the expectation for physicians to take the time and go over digitally provided information before a meeting to enhance its’ quality. Other perceived implications were the potential of resource-efficient avoidance of duplicate tests thus reducing expenditures for 2 (18%) patients and connecting further providers including hospitals, rehabilitation clinics, and medical specialists to improve care processes overall for 3 (27%) patients.

Certainly, I expect that we can have a more efficient conversation after providing information ahead of the appointment, rather than handing over a paper document to the doctor who starts reading and I start explaining what I actually understood.

Patient #06, male

Physicians saw positive implications for cross-sectoral care with regards to the possibility of faster communication between all parties involved, the economy of time in case of emergency or locum care, and second opinion cases. Besides the implied optimization potential for care processes and patient safety, utilizing PEPA was also considered to be an incentive to increase the transparency of documentation. With regards to aspects of health literacy, PEPA was not viewed as a promoting factor.

I don’t think patients who don’t have a medical background would have a better preparation for their treatment. My opinion.

Physician #15, female

### Nationwide Implementation

The majority (8/11, 73%) of the interviewed patients considered a PEPA implementation in Germany to be realistic in the near future. While some favored voluntary use, other patients (5/11, 45%) envisioned a lifelong use for the general population starting with the date of birth and covered by adequate legal regulation.

A more pessimistic view was shared when skepticism about the intent to implement was voiced by 3 (27%) patients or when obstacles for an implementation process within 5 to 10 years were anticipated by 2 of the 3 (67%) physicians. Concerns were general skepticism and presumed non-acceptance, data security, and privacy advocates, financing of the system, and missing IT infrastructure on the provider level.

Although both user groups anticipated data privacy concerns, they did not report having any themselves. They assumed data security comparable to online banking and misuse highly improbable. Expressing disbelief in the impact of their contribution, interviewees were concerned about a potential implementation of functionally reduced systems or multiple diverse systems. Patients only contemplated possible ways of financing PEPA.

I don’t see a pure on-top financing, but it would have to be through passing along incurred savings from physicians and health insurers.

Patient #06, male

Patients and physicians shared perspectives on old or impaired people’s abilities and willingness to manage online records. The lack of necessary skills or general interest and older age were assumed to be restricting factors among 6 (55%) of the patients and all 3 providers alike. Implementing a proxy regulation to remedy such circumstances was suggested by 2 (67%) of the physicians.

...very old people and people living in care facilities, or impaired people, certainly there would be someone else managing it for them, that’s a totally different thing.

Patient #05, female

Anticipating potential improvements for care processes for patients as well as for providers, the expectation was phrased that a nationwide implementation would make the use of a PEPA system obligatory for GPs. The physicians saw potential with regards to inter-provider communication or second opinion cases, but anticipated patients’ concerns about transparency, resistance from older colleagues and technical challenges where digital documentation is not used yet. Both groups of users considered integration into primary systems critical for interoperability.

It was an extra step. I had to leave my primary system and enter the internet. The software is good, but it was an extra step for me. I would integrate it otherwise it is not reasonable.

Physician #03, female

### Miscellanea

Users did not report burdening, uncomfortable emotional experiences referring to utilizing PEPA. Patients excluded emotional distress caused by PEPA content as they deemed a difference to paper documentation unlikely. However, they had clear ideas for scenarios in which distress could occur. Three (27%) of the patients considered a high volume of documentation being uploaded within a short period as a potentially stressful situation. Patients also weighed in on the influence of individual factors like age, reluctance to system changes or being less computer-savvy in general. While both groups of users again stressed the importance of provider-patient dialog to minimize potential emotional distress, 1 (33%) physician also saw a need to keep doctors’ notes inaccessible to patients in cases where those are essential for the treatment but meant to stay invisible to the patient. This view was shared by 1 (9%) patient.

In a thought experiment, interviewees were asked for their advice to a friend or family member who hypothetically would be offered a chance to use PEPA. All gave positive feedback and stated they could only recommend it. Five (45%) of the patients would advise careful consideration of provider access authorization and were aware of potential computer literacy and transparency concerns. Physicians would not advise against utilizing PEPA and see it as a solution to centrally available documentation. Again, they anticipated the citing of data privacy and security concerns. A clear positioning regarding a potential incremental value for patients, care processes or institutions was not provided.

The predominant motive for study participation of all interviewees was the intent to contribute to research progress and help others by providing feedback. Patients also ascribed their decision to participate to personal attitude, belief in the system, professional background, and personal interest. Physicians saw an opportunity to learn about a new development and to participate for the patient’s or the university’s benefit and felt motivated by their patient. Participants had varying perceptions about PEPA’s concept and their role during the trial period, broadly ranging from simple system tester to valuable study participant. However, all participants provided detailed insights into their experiences, interpretations, and perspectives referring to PEPA.

## Discussion

### Principal Results

This study evaluated reported experiences to understand which insights could be gained from utilizing PEPA. Supporting previous findings [3,31,32], results show that patients recognized benefits and felt enabled to participate more actively in their care. One of PEPA’s values was seen in the prompt sharing of digitally available documentation. Since accessing and engaging with documentation was simplified, PEPA functioned as a starting point for preparation and follow-up of appointments and related research. Patients added and read the documentation at their convenience, allowing them to view, review and contemplate content and its’ meaning before asking for additional explanations. This more active role implicated expectations for a transformed physician-patient interaction and care delivery. Naturally, patients felt that PEPA’s value was diminished where GPs chose not to participate.

Although the benefits of a central chronological documentation system were apparent to physicians, they did not use it to follow-up on patient history and mainly contributed by collecting and sharing data. Given the situation of working closely with a cancer patient they knew well, history details possibly were of lesser importance than they might have been with a different patient collective and outside of the study context. Physicians acknowledged the system’s potential to provide important information in emergency situations, locum care, and second opinion cases and to improve cross-sectoral communication. Nevertheless, they omitted a positioning regarding an incremental value for the physician-patient relationship or their health care organization. This reflects in postulating the integration into primary documentation systems as being crucial for interoperability and willingness to adopt PEPA.

All participating patients endorse implementing PEPA into standard care on a national level where availability of its various benefits is pending. They expect physicians to see the chances, not just obstacles and join modernization efforts thus fostering more effective, satisfying and genuinely collaborative care. Though they had diverse self-concepts or even doubts regarding their role and impact as study participants, all of them provided valuable input and shared the desire to purposefully contribute to research progress for the future benefit of others. This conveys the significance of including patients’ views and experiences in research projects.

Despite a thorough investigation and integration of user requirements [10,11], and exploration of challenges for cancer care coordination (14), utilizing PEPA was not an easy, self-explanatory concept for all participants. Depending on individual aptitudes and skills, deficits in understanding e-technology and PEPA’s functional concept became apparent. Though a longer usage period may contribute to a better understanding, a nationwide implementation would likely encounter similar circumstances to be addressed, both at the outset and continuously.

Both user groups anticipated concerns about data privacy but did not cite any themselves. While the concerns presumably recede into the background when people are confronted with severe illness and focus on optimized continuity of their care, a nationwide implementation most likely would encounter them. For the benefit of future users and a successful PEPA adoption, it appears advisable to address concerns and skills deficits in explanatory, educational efforts.

While patients see transparency as significant for the continuity of cross-sectoral care, physicians presume a lack of readiness among their colleagues. This points to a potential obstacle for health service modernization efforts inherent in current role concepts. However, to unfold its’ full potential and become a systemwide feature, PEPA’s socially challenging concept requires all parties involved to see its’ value and address a changing provider-patient relationship and transformed roles. A strategy of passively awaiting a change in traditional role concepts presumably will not suffice.

### Comparison to Previous Work

The findings in this study are supported by prior research showing patient empowerment effects through full health record access [33], patient benefits deriving from using a patient-controlled PHR [34], reservations regarding data privacy and the significance of the provider-patient relationship [35]. It demonstrates that patients with severe illness can utilize PEPA and manage their health documentation without experiencing related distress, provided constructive physician-patient communication is maintained. It can be assumed that further patient collectives would acknowledge the benefits and use of PEPA without distress as well. Nevertheless, concerns for older and impaired persons and corresponding support strategies are to be addressed by relevant politically responsible institutions.

### Strengths and Limitations

After implementing the prototype into a real-world care setting, exploring user experiences and perspectives was essential to understand potentially influential connotations regarding a future nationwide implementation and adequate refinements and went beyond an outcome-focused evaluation. To minimize research bias and to reduce the risk of losing relevant content, the analysis was guided by adequate methodological strategies. To indicate the typicality of observations, simple counts have been included where their support of the analysis can be expected and to meet potential notions of anecdotalism and exoticism, thus contributing to transparency.

However, some limitations must be declared for participant recruitment. PEPA was implemented for gastrointestinal cancer patients and their physicians for 3 months. The sample resulted from clinical practice and potentially was subject to selection bias. There was no control group for comparisons. Since the sample size was solely based on matters of feasibility, this resulted in a number of patients meeting the inclusion criteria which was too small to form a control group. Participants lost to the sample could not be substituted. A higher number of participants could have provided more diverse results. Though structural variance was given through age and sex, the small number of participants and short implementation period require cautious interpretation of all findings.

### Conclusions

Health care providers and patients alike can benefit substantially from ongoing digitalization efforts in the organization of German health care services. New skills will be needed on both sides to design and invigorate modern care processes and transformed roles. Decision makers and providers need to position themselves clearly and contribute towards closing modernization gaps. Committing to new concepts such as PEPA will be essential, and physicians need to sign on to them to make a nationwide implementation and utilization possible in the near future.

## Abbreviations

GP: general practitioner
INFOPAT: information technology for patient-oriented care
NCT: National Center for Tumor Diseases
PHR: personal electronic health record
